# Multi-format open-source weed image dataset for real-time weed identification in precision agriculture

**DOI:** 10.1016/j.dib.2023.109691

**Published:** 2023-10-18

**Authors:** Nitin Rai, Maria Villamil Mahecha, Annika Christensen, Jamison Quanbeck, Yu Zhang, Kirk Howatt, Michael Ostlie, Xin Sun

**Affiliations:** a Department of Agricultural and Biosystems Engineering, North Dakota State University, Fargo, ND 58102, USA; b Department of Electrical and Computer Engineering, Fargo, ND 58102, USA; c Department of Plant Sciences, North Dakota State University, Fargo, ND 58108, USA; d NDSU Carrington Research Extension Centre, 663 Hwy, 281 N, PO Box 219, Carrington, ND 58421-0219, USA

**Keywords:** Weed detection, Deep learning, Artificial intelligence, Weed species, Drone images, Greenhouse images

## Abstract

Weeds are considered obnoxious and a hindrance to crop yield. Due to their uneven spatial distribution pattern, a ground or aerial robot are deployed to spot spray herbicides. This herbicidal application depends entirely on the computer vision algorithms that assist with in-field weed identification prior to spot spraying. Therefore, to develop advanced computer vision algorithms, big data pertaining to agricultural weed dataset are required. In the past, public domain weed dataset have been released but mostly acquired using ground-based technologies. The dataset discussed in this paper is unique in that it incorporates data captured both from handheld camera and unmanned aerial system (UAS), thus catering to both ground-based and aerial-based weeding robots. This dataset comprises of 3,975 images featuring five different weed species commonly found in North Dakota: kochia *(Bassia scoparia)*, common ragweed *(Ambrosia artemisiifolia)*, horseweed *(Erigeron canadensis)*, redroot pigweed *(Amaranthus retroflexus)*, and waterhemp *(Amaranthus tuberculatus)*. These images have been meticulously annotated in various formats to facilitate the development and advancements of computer vision algorithms. Furthermore, various augmentation techniques have been applied to ensure that the dataset closely represents the real-world field conditions. Additionally, this dataset is open-source to assist precision weeding technologies for real-time in-field weed identification followed by herbicidal spot spraying application, ultimately contributing to more efficient and sustainable agricultural practices.

Specifications Table

**Table:** 

Subject	Precision Agriculture
Specific subject area	Weed detection, Weed localization, Convolutional neural network, Computer vision, and Deep learning.
Data format	Raw JPG with annotated weeds in formats including TXT, XML, and YAML.
Type of data	Raw images Multiple formats consisting of bounding-box annotations exported in multiple format such as, TXT, XML, and YAML.
Data collection	Greenhouse dataset was collected on multiple days and time of the day with varied levels of background and lighting conditions. Similar procedure was followed when collecting in-field data. The in-field data was collected using a DJI Phantom 4 Pro (V2.0). Several field parameters were considered while collecting the data to make it a best representation of the real world. These parameters were, different soil backgrounds, crop occlusion, unknown look-alike weeds and objects, shadow, and image blurring.
Data source location	1. Institution: North Dakota State University (NDSU) 2. City/Town/Region: North Dakota 3. Country: United States of America 4. Latitude and Longitude (with GPS Coordinates for the collected samples): a. NDSU-Greenhouse (46° 53’ 39.156” N, 96° 48’ 30.816” W) b. Agronomy Seed Farm in Casselton (46° 54’ 1.8” N, 97° 12’ 40.896” W) c. Carrington Research Extension Centre (CREC) (47° 22’ 25.7556” N, 99° 12’ 8.5032” W) d. Grand Farm (46˚ 43’10.3” N, -96˚ 49’41.5” W)
Data accessibility	Repository name: Mendeley Data Direct URL to data: https://data.mendeley.com/datasets/8kjcztbjz2/2

## 1 Value of the Data

• This dataset would be beneficial for researchers working within the deep learning field to develop computer vision-based weed identification techniques for in-field spot spraying applications.
• This dataset has the potential to be added to the custom datasets used by other researchers or users. This addition would enhance their datasets, leading to better algorithm development and improved generalization abilities.
• Due to the added advantage of multiple formats within this dataset, researchers would be able to deploy various deep learning models on-the-go for weed identification tasks, thereby eliminating the need to convert the dataset into specific model training formats.
• With the inclusion of two categories, aerial weeds and individual weeds, this dataset can assist both ground-based and aerial-based technologies in identifying and locating weeds for precise herbicidal application.

## 2 Objective

This dataset has been developed with both ground-based and aerial-based weed identification technologies in mind. The greenhouse images, along with clipped aerial images, would assist ground technologies in identifying weeds within a limited field of view. Meanwhile, the weed instances captured in high-resolution aerial images could help drone technologies locate weeds in a large field of view. A combination of both categories would advance computer vision models’ ability to extract weed information from complex backgrounds, thus enhancing their capability to generalize weed identification amongst crop plants in unseen locations. This dataset comprises multiple model training formats that can be used by researchers working on specific computer vision models for training purposes. The dataset presented in this paper explores the applicability of computer vision models not only for identifying specific weed species but also for locating them in preparation for spot spraying applications.

## 3 Data Description

### 3.1 Nature of in-field experiment

**Fig. 1:**
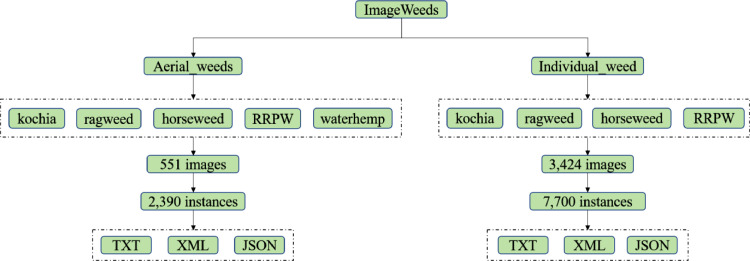
An overview of the dataset flowchart consisting of two dataset categories, aerial weeds and individual weed species exported in multiple formats.

The major objective of this dataset was to create weed categories that could assist ground as well as aerial weeding technologies for precise herbicidal spraying application. Therefore, after determining the end goal, the dataset was divided into two categories, aerial weeds and individual weeds (Fig. 1).

**Fig. 2:**
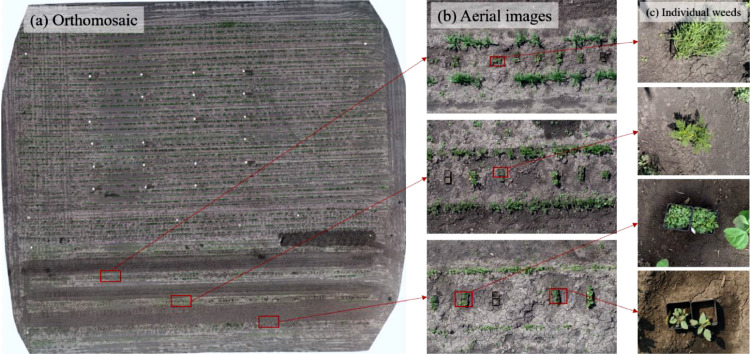
The orthomosaic of the experimental plot used to capture in-field images for further annotation, (a) orthomosaic created by flying UAS at 32 ft (∼9.7 m) displaying the nature of in-field experiment, (b) aerial images that were captured by flying UAS at an average altitude of 12 ft (∼3.7 m), and (c) individual weed species clipped from the aerial images captured in b.

The experimental plots for in-field data collection were designed as shown in the orthomosaic (Fig. 2a). To imitate real field conditions, multiple crops were placed side-by-side consisting of weeds in the centre (Fig. 2b). The aerial images were captured in multiple locations (Fig. 2b) out of which individual weeds were clipped (Fig. 2c). Similar plot design and image acquisition process was carried out in all the three locations.

## 4 Dataset Categories

Within the Mendeley dataset repository, two zipped files have been added. These two files are named Aerial_weeds.rar (A) and Individual_Weed.rar (I). Within Aerial_Weeds file, there are two folders named “images” and “labels.” The “images” folder consists of high-resolution aerial images in JPG format, while the “labels” folder contains three different subfolders, each dedicated to a specific format: JSON, TXT, and XML. Similar organizational criteria have been applied to the “Individual_weed” category as well. For individual weeds, images and labels of each individual weed species are stored within a respective class of weed species.

**Fig. 3:**
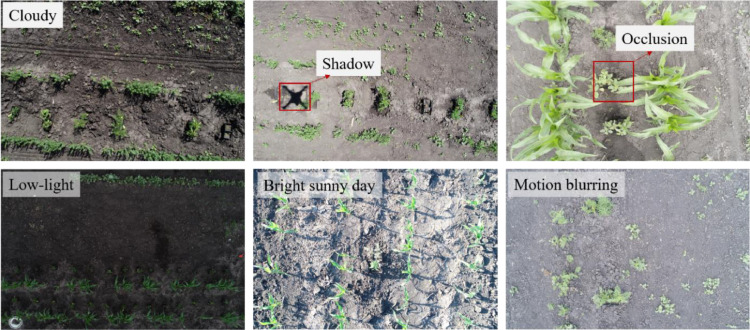
Sample images of Aerial Weeds dataset along with several in-field parameters that were added while collecting the dataset in 2 locations. These in-field parameters were, (a) cloudy environment, (b) drone shadow on a weed specie, (c) weeds occluded due to corn crops, (d) dark environment using low-light settings, (e) images captured around noon hours when the sun would be overhead, and (f) motion blurring caused due to drone speed.

The aerial weeds dataset was captured using off-the-shelf unmanned aerial system (UAS), the DJI Phantom 4 Pro (V2.0), in three different locations: Casselton, Carrington, and Grand Farm. The UAS was flown at an average altitude of 12 ft (∼ 3.7 m) at varied speed in multiple environmental settings. The selection of this altitude was a result of careful consideration to ensure the acquisition of distinct weed images and features for DL applications. The images in Casselton were captured during the summer season, specifically from late May to late June 2021, while in Carrington, the data collection occurred from mid-July to late August of 2021. In Grand Farm, data collection occurred from mid-August to late September 2022. The choice of a two-year time frame was deliberate, aiming to incorporate both temporal and location-based diversity into the dataset for robust data generation. chosen so that temporal as well as location wise diversity could be adopted for data generation. The aerial images were captured at a resolution of 5,472 × 3,648 pixels in JPG format and encompassed a range of diverse in-field settings. A sample of the aerial images captured in multiple locations is depicted in Fig. 3. Furthermore, approximately 550 images were collected for which manual annotations of weeds were created, resulting in over 2,390 individual instances. Table 1 provides specific details on these instances, including manually drawn bounding-boxes, categorized into five distinct weed species classes.

**Table 1:** Number of images and instances (annotations) for each weed species in both the categories of the dataset.

Weeds	Aerial_weeds (A)	
	Total images	Total instances
Kochia	551	600
Horseweed		400
Ragweed		650
RRPW		320
Waterhemp		420
Total ^¥^	—	2,390
	Individual_weed (I)	
Kochia	1,150	2,600
Horseweed	1,032	1,700
Ragweed	878	2,000
RRPW	364	1,400
Total ^€^	3,424	7,700
^(¥+€)^ Total number of sets in the repository	3,975	10,090

**Fig. 4:**
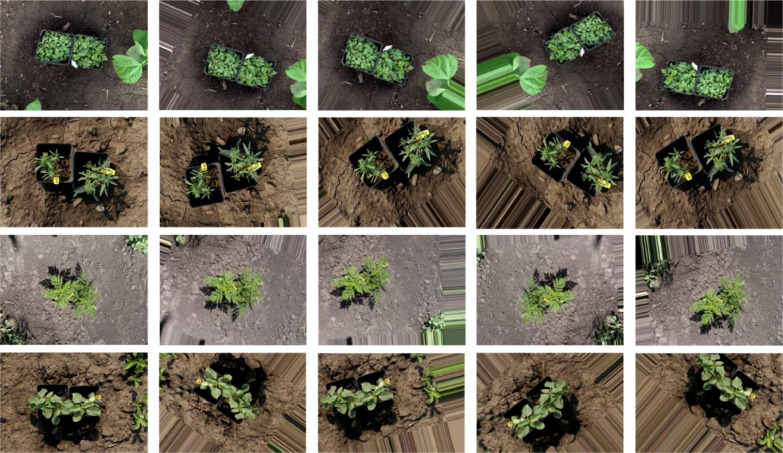
Individual weed specie consisting of original images as clipped (1^st^ column) and corresponding augmented types (columns 2–5).

To create dataset for individual weeds, specific classes of each weed species were extracted from aerial images and subsequently manually annotated in multiple formats. In addition, to these images, several greenhouse images, captured using a handheld Canon 90D camera, were included to introduce diversity within the training set. In total, over 3,424 images were manually annotated, resulting in the generation of 7,700 instances covering four weed classes (as detailed in Table 1). Fig. 4 showcases sample images of the clipped weed species that were annotated and exported in multiple data formats.

## 5 Experimental Design, Materials and Methods

### 5.1 Dataset augmentation

**Fig. 5:**
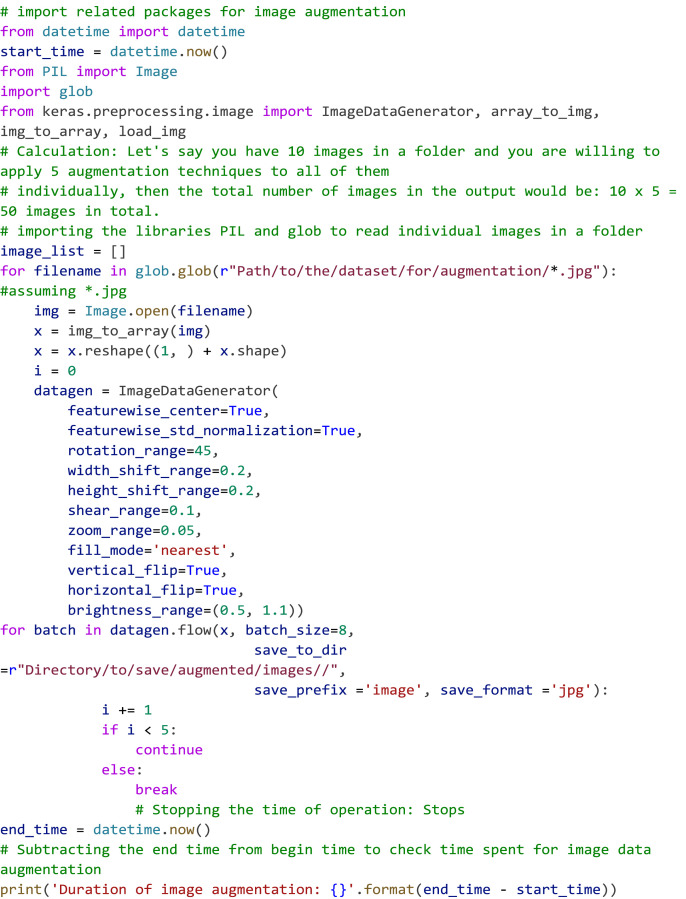
Code snippet showcasing various data augmentation techniques applied on the individual weed category of the dataset.

To enhance the volume and diversity of the dataset [1], data augmentation techniques were applied on the individual weed category. The original number of images for kochia, horseweed, ragweed, and RRPW were 785, 448, 355, and 115, respectively. These images underwent further augmentation and the final output images are outlined in Table 1. Additionally, APIs from Keras Image data generator were employed to execute various augmentation types, including feature wise centre, shear range, zoom range, horizontal flip, rotate, shear etc., all within a Python platform. The specific versions used for pre-processing and conducting data augmentation are elaborated in Table 2. (Fig. 5)

**Table 2:** Python libraries and its respective versions used to perform image pre-processing and data augmentation.

Frameworks	Versions
Python	3.8.5
PIL	8.0.1
Glob	0.7
Keras	2.6.0

## 6 Dataset annotation

**Fig. 6:**
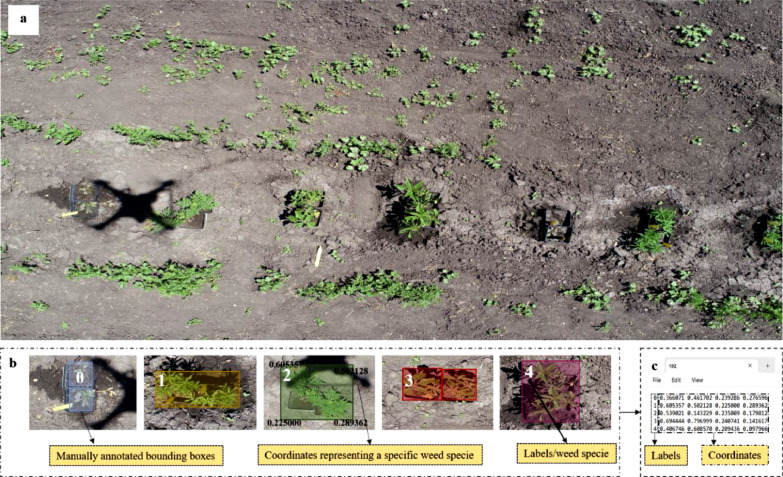
Manual annotation of the image data consists of three steps, (a) acquiring the aerial image, (b) manually annotating each class of weed species, and (c) exporting a text file consisting of labels and coordinates.

After the images were augmented, both categories of the dataset were annotated and exported using the LabelImg [2] tool. LabelImg is an open-source software that can be used to annotate objects of interest and export data in multiple formats for training various deep learning models. Fig. 6 showcases a sample of aerial image that was imported into the software and subsequently annotated manually with weed species (Fig. 6b). The software itself defines the four coordinates of the annotated weed species. Finally, after manual annotation, a text file (*.txt) was exported containing information organized in two columns: labels and coordinates (Fig. 6c). The labels correspond to specific classes of weed species with their respective coordinate in the second column. The TXT format thus exported has been widely adopted to train several state-of-the-art YOLO models based on COCO dataset [3].

## 7 Exporting the Dataset in Multiple Formats

**Fig. 7:**
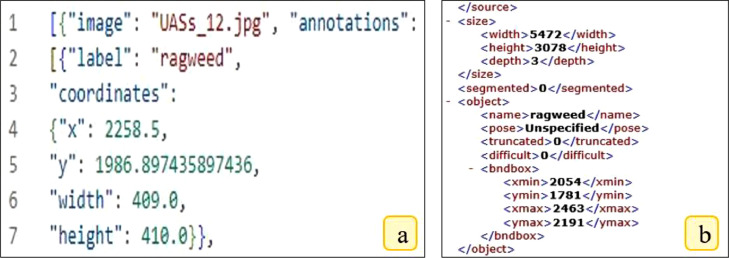
JSON and XML formats as exported using the LabelImg tool. In these formats, (a) JSON format, and (b) XML format as exported using the LabelImg tool.

Following the output discussed in the previous section (*.txt format), JSON (JavaScript Object Notation) and XML (Extensible Markup Language) formats were also exported using the same LabelImg software. Fig. 7 (a & b) showcases the exported output of these sample formats. The JSON format comprises five parameters that defines the annotated object of interest. In Fig. 7a, ragweed has been annotated with the coordinates x, y, width, and height. Similarly, Fig. 7b presents the XML format, which includes the image resolution followed by four coordinates defining the object of interest, namely, ragweed. The following formats have been used in creating PASCAL VOC dataset to train various detection and segmentation models [4].

## 8 Limitations

This dataset was captured while keeping all the field parameters in mind. However, this dataset does not include images that have been distorted due to the downward gust of wind pressure created by the drone while descending to capture images. Typically, the downward wind pressure can alter the physiological appearance of the weeds, making it challenging for deep learning algorithms to detect and locate the weed species, potentially resulting in incorrect identification and improper spot spraying. This aspect of data acquisition has not been considered either during in-field image capturing procedure or the pre-processing steps.

## Ethics Statement

This dataset does not involve experiments on humans or animals nor does it tend to collect data from any social media platforms.

## CRediT authorship contribution statement

**Nitin Rai:** Conceptualization, Methodology, Software, Investigation, Writing – original draft. **Maria Villamil Mahecha:** Data curation. **Annika Christensen:** Data curation. **Jamison Quanbeck:** Data curation. **Yu Zhang:** Supervision, Project administration. **Kirk Howatt:** Supervision, Project administration. **Michael Ostlie:** Supervision, Project administration. **Xin Sun:** Writing – review & editing, Supervision, Project administration, Funding acquisition.

## Data Availability

ImageWeeds: An Image dataset consisting of weeds in multiple formats to advance computer vision algorithms for real-time weed identification and spot spraying application (Original data) (Mendeley Data)
